# Reply to Lee, S.Y. Comment on “Sung et al. Body Fat Reduction Effect of *Bifidobacterium breve* B-3: A Randomized, Double-Blind, Placebo Comparative Clinical Trial. *Nutrients* 2023, *15*, 28”

**DOI:** 10.3390/nu15051094

**Published:** 2023-02-22

**Authors:** Hyun Kyung Sung, Sang Jun Youn, Yong Choi, Sang Won Eun, Seon Mi Shin

**Affiliations:** 1 Department of Pediatrics, College of Korean Medicine, Semyung University, Jecheon-si 27136, Republic of Korea; 2 RnBS Corp., Seoul 06032, Republic of Korea; 3 Daehan Chemtech Co., Ltd., Seoul 01811, Republic of Korea; 4 Department of Internal Medicine, College of Korean Medicine, Semyung University, Jecheon-si 27136, Republic of Korea

Thank you kindly for your interest in and opinion [1] on our research [2].

In this clinical trial, the intake of probiotics and lactic acid bacteria was prohibited during the study period, and participants who used them were excluded. Additionally, if changes in intestinal flora were analysed, the mechanism of body fat reduction could have been elucidated; however, the research team agreed that the intestinal microbiome test would not be conducted.

Bilsborough et al. [3] assessed reliability using a test–retest methodology to compare the precision of two dual-energy X-ray absorptiometry (DEXA) devices in highly trained Australian football players. They found a reliability of 0.3% (coefficient of variation) for lean body mass and 2.5% (coefficient of variation) for body fat mass and body fat percentage after performing two scans on each DEXA device. To assess test–retest reliability, the athletes were instructed to follow standard protocols of food and fluid intake to ensure optimal hydration before each scan. Therefore, it can be said that reliability was assessed in the same physical conditions as far as possible, without changes in body fat mass and lean body mass caused by other interventions.

This trial aimed to confirm the effect of reducing body fat mass after taking investigational foods in overweight or obese adults. For test–retest reliability, repeated DEXA measurements should be conducted under similar conditions. However, in this study, measurements were conducted before and after intervention; therefore, it is meaningless to report reliability using the coefficient of variation, which represents DEXA precision, because the subjects’ body fat mass and lean body mass had changed due to investigational food intake.

The intake determination data are as follows. Five-week-old male C7BL/6J mice were fed approximately 166 g of a high-fat diet mixed with 108 or 109 CFU/d of *Bifidobacterium breve* B-3 for an 8-week study period. Anti-obesity efficacy was confirmed, and there was no report of toxicity [4]. Furthermore, at Tokyo Medical and Health University in Japan, individuals with a high body mass index were given food containing *B. brev* B-3 (5.0 × 10^9^ CFU/d), and the effect on body weight, body fat, and blood parameters was assessed. A decrease in body fat mass was confirmed in the *B. brev* B-3 intake group [5]. Accordingly, the daily intake of the test substance *B. brev* B-3 was set at 5 billion CFU/d considering the human application test results of similar raw materials and subject safety.

Despite some studies reporting that vaccination can affect weight, there are more reports on weight gain related to social distancing—which was more common than vaccination—due to COVID-19 [6,7,8]. Rather, the possibility of weight gain is higher in a pandemic situation; hence, the fact that there was weight loss in this study conducted during this period can be considered a remarkable achievement.

In this study, as per the protocol, per-protocol (PP) analysis was performed for efficacy evaluation as the purpose was to estimate the true efficacy of the intervention on subjects who have completed the trial.

More research is needed on the mechanism of body fat reduction. Moreover, this study was conducted by dividing subjects into two groups through random assignment, and there was no difference between the groups in terms of dietary habits or amount of exercise performed at the beginning of the study (Table 1).

The limitations to this study were mentioned in the published paper [2].

## Figures and Tables

**Table 1:** Changes in physical activity and dietary habits (PPS).

		BB-3 Group (*N* = 42)	Placebo Group (*N* = 41)	*p*-Value *
IPAQ Total Physical activity (MET-min/week)	V2	2401.50 ± 1955.78	2848.59 ± 3562.14	0.6819 ^1^
	V5	4151.02 ± 5655.05	2808.20 ± 3294.04	0.3164 ^1^
	V5 − V2	1749.52 ± 4582.37	−40.39 ± 2645.99	0.3412 ^1^
IPAQ Total Energy expenditure (kcal/week)	V2	3065.91 ± 2694.03	3653.76 ± 4955.91	0.7327 ^1^
	V5	5257.72 ± 7531.41	3509.73 ± 4546.55	0.3275 ^1^
	V5 − V2	2191.81 ± 5915.85	−144.02 ± 3317.14	0.3458 ^1^
Dietary survey (kcal)	V2	1883.56 ± 573.85	1827.53 ± 657.96	0.5447 ^1^
	V5	1811.72 ± 440.19	1721.49 ± 545.25	0.2006 ^1^
	V5 − V2	−71.84 ± 579.75	−106.03 ± 704.08	0.8734 ^1^

* Compared between group; ^1^ *p*-value for the Wilcoxon rank sum test; The International Physical Activity Questionnaire (IPAQ) was used to assess physical activity, while the 24 h dietary recall method was used to assess dietary habits.
